# Bioresource Nutrient Recycling in the Rice–Wheat Cropping System: Cornerstone of Organic Agriculture

**DOI:** 10.3390/plants10112323

**Published:** 2021-10-28

**Authors:** Saba Nazir, Qamar uz Zaman, Asim Abbasi, Nayab Komal, Umair Riaz, Kamran Ashraf, Nabeel Ahmad, Shweta Agarwal, Rabiya Nasir, Yinglong Chen

**Affiliations:** 1 Department of Environmental Sciences, The University of Lahore, Lahore 54590, Pakistan; sabawarraich989@gmail.com (S.N.); nayabkomal5@gmail.com (N.K.); rabiya.nasir@envs.uol.edu.pk (R.N.); 2 Department of Zoology, Punjab Group of College, University of Central Punjab, Bahawalpur 63100, Pakistan; asimuaf95@gmail.com; 3 Soil and Water Testing Laboratory for Research, Agriculture Department, Government of Punjab, Bahawalpur 63100, Pakistan; umairbwp3@gmail.com; 4 Department of Food Science and Nutrition, Faisalabad Sahiwal Campus, Government College University, Sahiwal 57000, Pakistan; kamran2417@gmail.com; 5 Department of Public Health, Torrens University, Melbourne 3000, Australia; nabeel.ahamd@tulc.torrens.edu.au; 6 Department of Business, Torrens University, Melbourne 3000, Australia; shweta.agarwal@torrens.edu.au; 7 The UWA Institute of Agriculture, School of Agriculture and Environment, The University of Western Australia, Perth 6009, Australia; 8 Institute of Soil and Water Conservation, Chinese Academy of Sciences, Northwest A&F University, Yangling 712100, China

**Keywords:** biological functions, diverse farming approaches, enzymatic activities, residue management, soil health, sustainability

## Abstract

This study evaluated the impact of conventional practices (fertilizer alone) and diverse farming approaches (such as green manuring, farmyard manure application, rice-residue incorporation, residue mulching, residue removal and residue burning) on soil attributes. A total of thirty-five farm sites were selected, with five sites (replications) for each farming approach system, which were used over the past three years in the study farms. Characterization of rice residues of all cultivars, green manure crop (sesbenia: *Sesbania sesban*) and decomposed farmyard manure samples showed differential behaviours for macronutrients and micronutrients. Continuous application of inorganic fertilizers significantly influenced soil attributes, especially electrical conductivity, nutrient contents, bacterial and fungal population and soil enzymatic attributes. The crop residue treatments favourably influenced the soil parameters over the control. Crop residue incorporation or burning significantly increased soil available potassium, microbial biomass, enzymatic activities and organic carbon when compared with applications of chemical fertilizer alone, while total nitrogen content was increased by residue incorporation. However, green manuring and farmyard manure applications showed inferior responses compared with residue management treatment. It is therefore recommended that bioresources should be managed properly to warrant improvements in soil properties, nutrient recycling and the sustainability for crop productivity, in order to achieve sustainable development goals for climate action.

## 1. Introduction

The growing demand for food in developing countries has led to tremendous increases in food production around the world. Hence, agro-based activities represent profitable businesses, both in developing as well as developed countries [1,2,3]. Soil health is an ancient and ubiquitous concept [4,5,6,7]. It would be impossible for us to meet the growing demands for food, feed, fibre, and fuel if we could not maintain soil health properly, instead we would see increased surface disturbance, increased land erosion and reduced plant diversity due to intensive agriculture [8,9]. Soil health depletion can often be self-reinforced, as low-quality soils (having unsustainable agricultural practices) produce low-quality biomass, which in turn, results in low-quality manure. The low-quality manure immobilizes nutrients in the soil and thus perpetuates a cycle that reduces soil health [1,9].

Chemical fertilizers not only improve crop production by supplying more nutrients to the soil for plant uptake, but they also affect the soil’s physical, chemical, and biological properties positively or negatively [3]. These soil attributes combined maintain soil health and improve crop growth and can be evaluated by soil quality [10]. Long-term and intensive application of chemical fertilizers influences soil physicochemical attributes, such as texture, compaction, infiltration rate, seepage, hydraulic conductivity, soil porosity, bulk density, nutrients status, cation exchange capacity, electrical conductivity, pH, and soil microbial-community change [11].

Crop residue management is a widely accepted practice for improving soil physical, chemical, and biological functions, including soil microbe communities and arbuscular mycorrhizal fungi. Maintenance of crop residue cover on the soil surface benefits belowground food webs and processes, and improves abundance and diversity of soil bacterial communities, including beneficial microbes such as *Pseudomonas, Burkholderiales* and *Rhizobiales,* which have plant-growth promoting capacities [12,13], and arbuscular mycorrhizal fungi with beneficial effects on crop yield and biocontrol [6,7]. However, decomposition of crop residues has both positive and negative impacts on crop production [14]. The negative effects of allelochemicals from crop residues on crop growth can be adjusted by crop residue returning management [15]. Soil management with crop residues has a wide range of advantages in improving soil health and crop production, including residue decomposition, soil erosion control, nutrient recycling and availability to plants, control of weed pests, and various conservation practices related to tillage for maximizing crop yields [9,16,17,18,19].

The use of farmyard manure (FYM) alone as a substitute to inorganic fertilizer is not enough to maintain the present levels of crop productivity of high-yielding varieties [20]. Emerging evidence has indicated that integrated soil-fertility management, involving the judicious use of combined organic and inorganic resources, is a feasible approach to overcome soil fertility constraints [21]. In the modern days of agricultural science, crop rotation and green manuring (GM) offer technologies able to achieve sustainable production efficiently. One of the options to maintain sustainability in agriculture is to add GM into the farmland to increase soil organic matter (OM) content through restoring soil quality (especially in tropical soils) and reclaiming degraded soil [22,23]. This practice is eco-friendly, nonpolluting, and nondegrading to soil, water and air [24,25,26,27].

Farmers in developing countries usually focus on chemical-based agriculture through excessive use of chemical-based fertilizers, the burning of crop residues and limited use of organic amendments, thereby severely affecting soil health. In many rural areas in Punjab, Pakistan, intensive crop production combined with removal or burning of crop residues has depleted agricultural soils, jeopardizing their productive capacity and ability to meet the needs of future generations. Holistic production-management systems that promote and enhance agroecosystem health are necessary, in order to protect our soils while maintaining high productive capacities contributing to ecological, economic and social sustainability. Thus, the present 3-year repeated study aims to develop a better understanding of low-cost, ecofriendly, nutrient-management technologies that involve recycling of locally available bioresources and sustaining crop productivity and soil health in the intensive agricultural systems in Pakistan.

## 2. Results

### 2.1. Nutrient Concentration in Farmyard Manure Samples

Data regarding the nutrient concentrations in the farmyard manure samples (composted for one year before use) collected from the various study area showed variation. A variety of primary and secondary nutrients, micronutrients and trace elements were present in all samples of farmyard manure. For primary nutrients, concentrations of nitrogen (0.91 ± 0.04%), phosphorous (0.36 ± 0.02%) and potassium (0.89 ± 0.05%) were noticeable. However, for secondary nutrients, the concentration of calcium (0.90 ± 0.03%), magnesium (0.20 ± 0.04%) and sulphur (0.02 ± 0.03%) were observed in the farmyard manure samples. Regarding the micronutrients and trace elements, concentrations of sodium (0.09 ± 0.03%), zinc (56 ± 1.23 ppm), iron (140.30 ± 3.42 ppm), boron (2.30 ± 0.97%), copper (2.80 ± 1.11%) and manganese (69.00 ± 1.32) were observed (Table 1).

### 2.2. Nutrient Concentration in Sesbania Samples

The findings of the current study revealed that the nutrients’ concentrations in the sesbania samples collected from various study areas showed variation. A variety of primary and secondary nutrients, micronutrients and trace elements were present in all samples of sesbania. For primary nutrient concentrations, nitrogen (3.32 ± 1.04%), phosphorous (0.73 ± 0.09), and potassium (1.32 ± 0.15%) were observed. However, for secondary nutrients, concentrations of calcium (1.34 ± 0.13%), magnesium (208.00 ± 3.74%) and sulphur (0.20 ± 0.12%) were observed in the sesbania samples (Table 1).

### 2.3. Elemental Concentration in Rice Residue Samples

**Figure 1:**
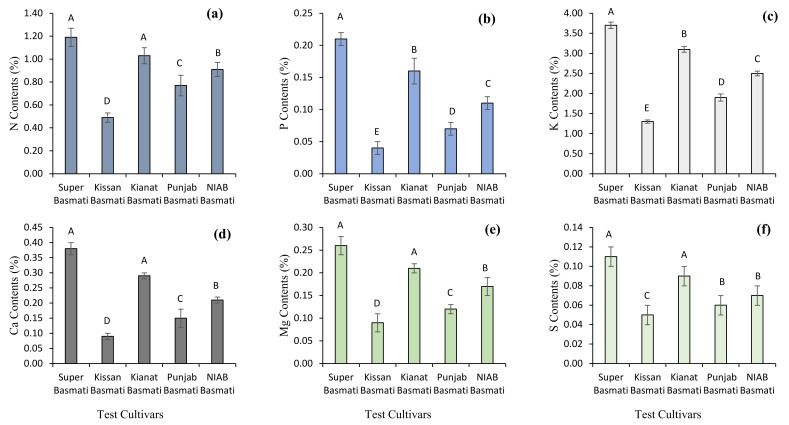
Primary and secondary nutrient concentrations [nitrogen contents (**a**), phosphorous content (**b**), potassium contents (**c**), calcium contents (**d**), magnesium contents (**e**), and sulphur contents (**f**)] in the rice residue samples. Bar data are means ± standard error (*n* = 3). In each graph, bar data with different letters indicate statistically significant difference at *p* ≤ 0.05.

**Figure 2:**
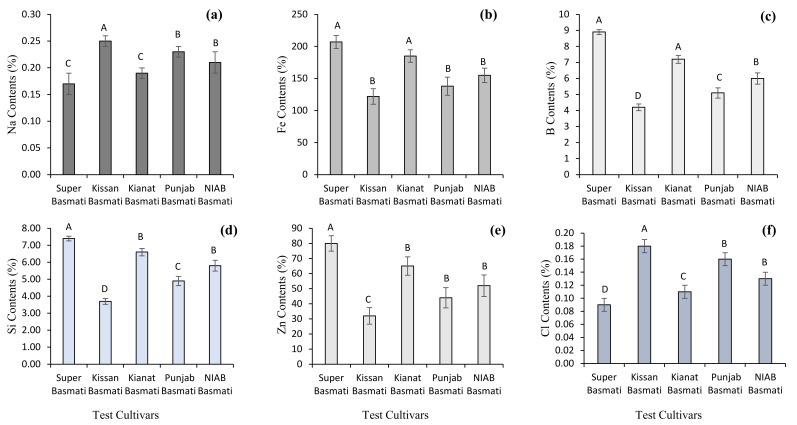
Micronutrients and trace element concentrations [sodium contents (**a**), iron contents (**b**), boron contents (**c**), silicon contents (**d**), zinc contents (**e**), and chloride contents (**f**)] in the rice residue samples; Bar data are means ± standard error (*n* = 3). In each graph, bar data with different letters indicate statistically significant difference at *p* ≤ 0.05.

The findings of the current study revealed that significant variation existed among the primary and secondary nutrient concentrations in straw residues of basmati rice cultivars. For primary nutrient concentrations, the results of ICP-MS showed that maximum nitrogen (1.09%), phosphorous (0.21%) and potassium (3.70%) concentrations were observed in the rice straw samples of the Super Basmati cultivar, followed by the concentrations in Kianat Basmati. Minimum nitrogen (0.49%), phosphorous (0.04%) and potassium (1.30%) concentrations were observed in the straw residues of Kissan Basmati. For primary nutrients’ results, maximum calcium (0.38%), magnesium (0.26%) and sulphur (0.11%) levels were observed in the rice straw samples of the Super Basmati cultivar, followed by the levels in Kianat Basmati. Minimum calcium (0.09%), magnesium (0.09%) and sulphur (0.15%) concentrations were observed in the straw residues of Kissan Basmati (Figure 1 and Figure 2). Significant variation was observed among the micronutrient and trace element concentrations in the straw residues of Basmati rice cultivars. Maximum sodium (0.25 mg g^−1^) and chloride (0.18 mg g^−1^) concentrations were found in Kissan Basmati. Iron (207.00 mg g^−1^), boron (8.9 mg g^−1^), silicon (7.40 mg g^−1^) and zinc (80.00 mg g^−1^) concentrations were noticed in straw residues of Super Basmati, while minimum sodium (0.17 ppm) and chloride (0.09 mg g^−1^) concentrations were found in Super Basmati. Iron (122.00 mg g^−1^), boron (4.2 mg g^−1^), silicon (3.70 mg g^−1^) and zinc (32.00 mg g^−1^) concentrations were observed in the straw residues of Kissan Basmati (Figure 2).

### 2.4. Diverse Farming Approaches and Soil Health

Results for physicochemical and nutrient contents of various samples collected before and after practicing the diverse farming approaches showed large variation. Soil sand contents (before treatment ranged from 49.45 to 49.52% and after treatment ranged from 49.36 to 49.65%), silt contents (before 28.44 to 28.50%, and after 28.91 to 28.91%), clay contents (before 19.33 to 19.42%, and after 19.24 to 19.66%) and soil bulk density (before 0.88 to 0.92 g/cm^3^, and after 0.81 to 0.92 g/cm^3^) showed large variation among the farming approaches.

**Figure 3:**
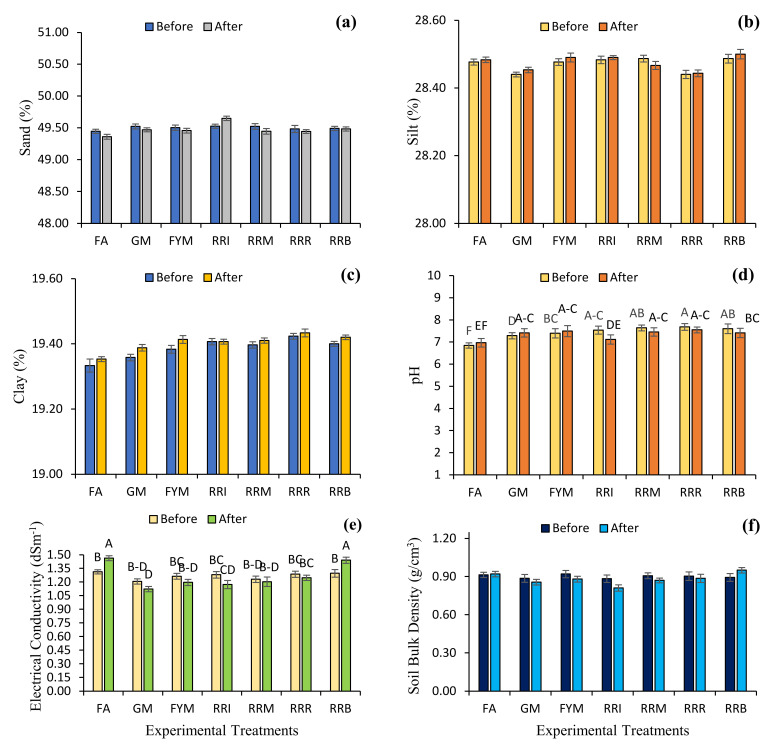
Effect of diverse farming approaches on the physicochemical attributes [sand contents (**a**), silt contents (**b**), clay contents (**c**), pH (**d**), electrical conductivity (**e**), and bulk density (**f**)] of soil samples by fertilizer application (FA); green manuring (GM); farmyard manure application (FYM); rice-residue incorporation (RRI); rice-residue mulching (RRM); rice-residue removing (RRR) and rice-residue burning (RRB). Bar data are means ± standard error (*n* = 3). In each graph, bar data with different letters indicate statistically significant difference at *p* ≤ 0.05.

**Figure 4:**
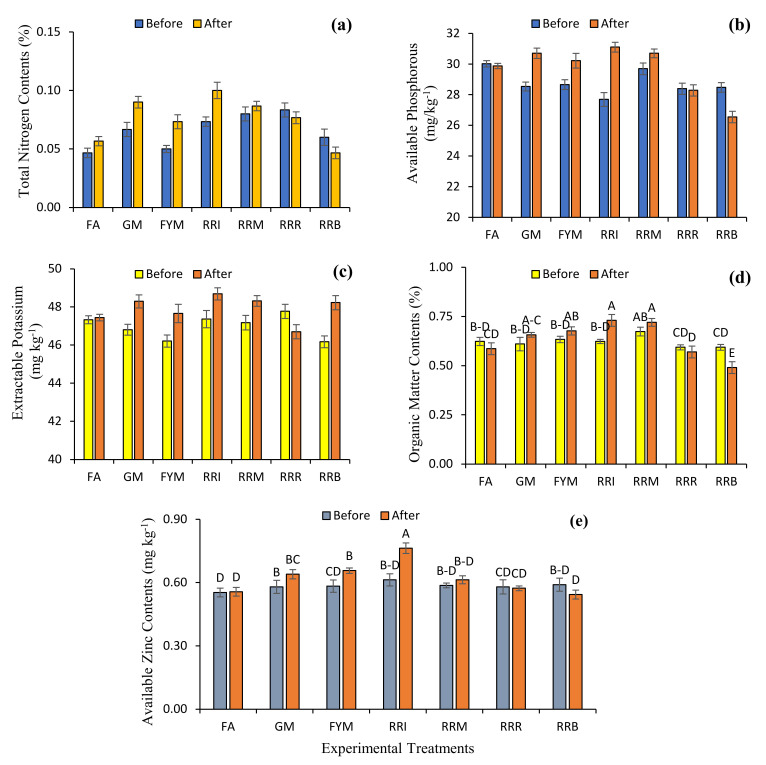
Effect of diverse farming approaches on the nutrient contents [total nitrogen contents (**a**), available phosphorous (**b**), extractable potassium (**c**), organic matter (**d**), and available zinc contents (**e**)] of soil samples by fertilizer application (FA); green manuring (GM); farmyard manure application (FYM); rice-residue incorporation (RRI); rice-residue mulching (RRM); rice-residue removing (RRR) and rice-residue burning (RRB). Bar data are means ± standard error (*n* = 3). In each graph, bar data with different letters indicate statistically significant difference at *p* ≤ 0.05.

Soil pH, EC and OM from different treatment sites with diverse farming approaches showed variation, before treatment soil pH ranged from 6.84 to 7.68, and after ranged from 6.97 to 7.55, before treatment EC ranged from 1.23 to 1.31 dS m^−1^, and after ranged from 1.17 to 1.46 dS m^−1^, and before treatment OM ranged from 0.59 to 0.67% and after ranged from 0.59 to 0.73%. Maximum pH (7.68) was observed in the soil samples collected before the removal of rice residues, followed by pH before rice-residue mulching (7.64). Maximum soil EC (1.46 dS m^−1^) was observed in the soil samples collected after the fertilizer application, followed by after rice-residue burning (1.44 dS m^−1^). Maximum soil OM (0.73%) was observed in the soil samples collected after the rice-residue incorporation, followed by after rice-residue mulching (0.72%) (Figure 3 and Figure 4). By performing practices such as application of fertilizer, green manuring and farmyard manure application, soil pH was increased by 1.85, 1.78 and 1.40%, respectively. However, rice-residue management interventions such as rice-residue incorporation, rice-residue mulching, rice-residue removal and rice-residue burning reduced soil pH 5.61, 2.40, 1.74 and 2.50% away from neutral, respectively. EC values after fertilizer application and rice-residue burning were increased by 11.42 and 11.05%, respectively. However, green manuring, farmyard manure application, rice-residue incorporation, rice-residue mulching and rice-residue removal decreased soil EC by 6.91, 5.28, 8.33 and 2.17 and 3.11%, respectively. Rice-residue removing and rice-residue burning decreased the soil OM by 5.88, 3.93 and 17.42%, respectively. However, soil OM values after the treatments of green manuring, farmyard manure application, rice-residue incorporation and rice-residue mulching were increased 7.65, 6.84, 17.11 and 6.93%, respectively (Table 2).

Nutrient contents in soils following the diverse farming approaches showed variation: available nitrogen before treatment ranged from 0.05 to 0.08%, and after treatment ranged from 0.05 to 0.10%, available phosphorous before treatment ranged from 28.39 to 30.02 mg kg^−1^, and after ranged from 26.66 to 31.11 mg kg^−1^, extractable potassium before treatment ranged from 46.18 to 47.77 mg kg^−1^, and after treatment ranged from 46.70 to 48.69 mg kg^−1^, and available Zn before treatment ranged from 0.55 to 0.61 mg kg^−1^, and after ranged from 0.54 to 0.71 mg kg^−1^ (Figure 4). Rice-residue removal and rice-residue burning decreased the total nitrogen contents by 8.00 and 22.22%, respectively. However, total available nitrogen concentrations in the treatments of fertilizer application, green manuring, farmyard manure application, rice-residue incorporation and rice-residue mulching were increased by 21.43, 35.00, 46.67, 36.36 and 8.33%, respectively. In the cases of available phosphorous concentrations, fertilizer application, rice-residue removing and rice-residue burning, concentrations were decreased by 0.47, 0.38 and 6.79%, respectively. However, green manuring, farmyard manure application, rice-residue incorporation and rice-residue mulching increased available phosphorous concentrations by 7.59, 5.43, 12.34 and 3.39%, respectively. Extractable potassium concentration following the rice-residue removal treatment was decreased by 2.24%. However, extractable potassium concentration following fertilizer application, green manuring, farmyard manure application, rice-residue incorporation, rice-residue mulching and rice-residue burning treatments was increased by 0.25, 3.18, 3.13, 2.79, 2.42 and 4.44%, respectively. Available Zn concentrations following rice-residue removing and rice-residue burning treatments were decreased by 1.15 and 7.91%, respectively. However, available Zn concentrations following fertilizer application, green manuring, farmyard manure application, rice-residue incorporation and rice-residue mulching treatments were increased by 0.60, 10.34, 12.57, 24.46 and 4.55%, respectively (Table 2).

**Figure 5:**
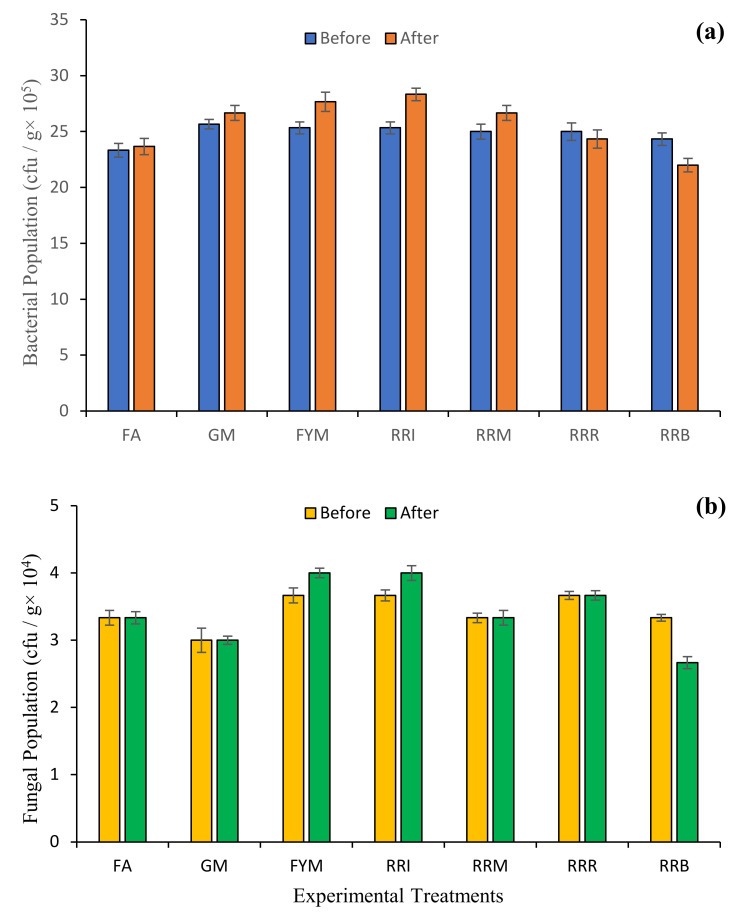
Effect of diverse farming approaches on the biological attributes [bacterial population (**a**) and fungal population (**b**)] of soil samples by fertilizer application (FA); green manuring (GM); farmyard manure application (FYM); rice-residue incorporation (RRI); rice-residue mulching (RRM); rice-residue removing (RRR) and rice-residue burning (RRB). Bar data are means ± standard error (*n* = 3).

Results for biological attributes of various samples collected before and after practicing the diverse farming approaches showed differential variation. Soil bacterial population before treatment ranged from 23.33 to 25.67 cfu/g × 10^5^, and after treatment ranged from 22.00 to 28.33 cfu/g × 10^5^. Soil fungal population before treatment ranged from 3.00 to 3.67 cfu/g × 10^4^, and after ranged from 2.67 to 4.00 cfu/g × 10^4^. These parameters differed among the treatments (Figure 5).

**Figure 6:**
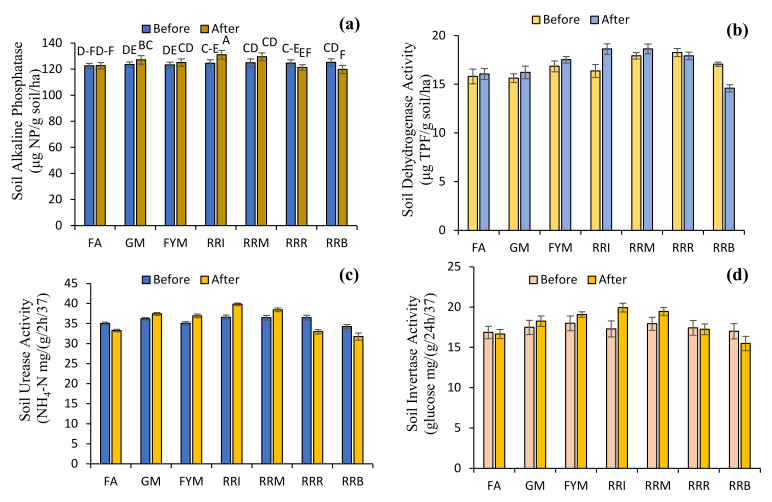

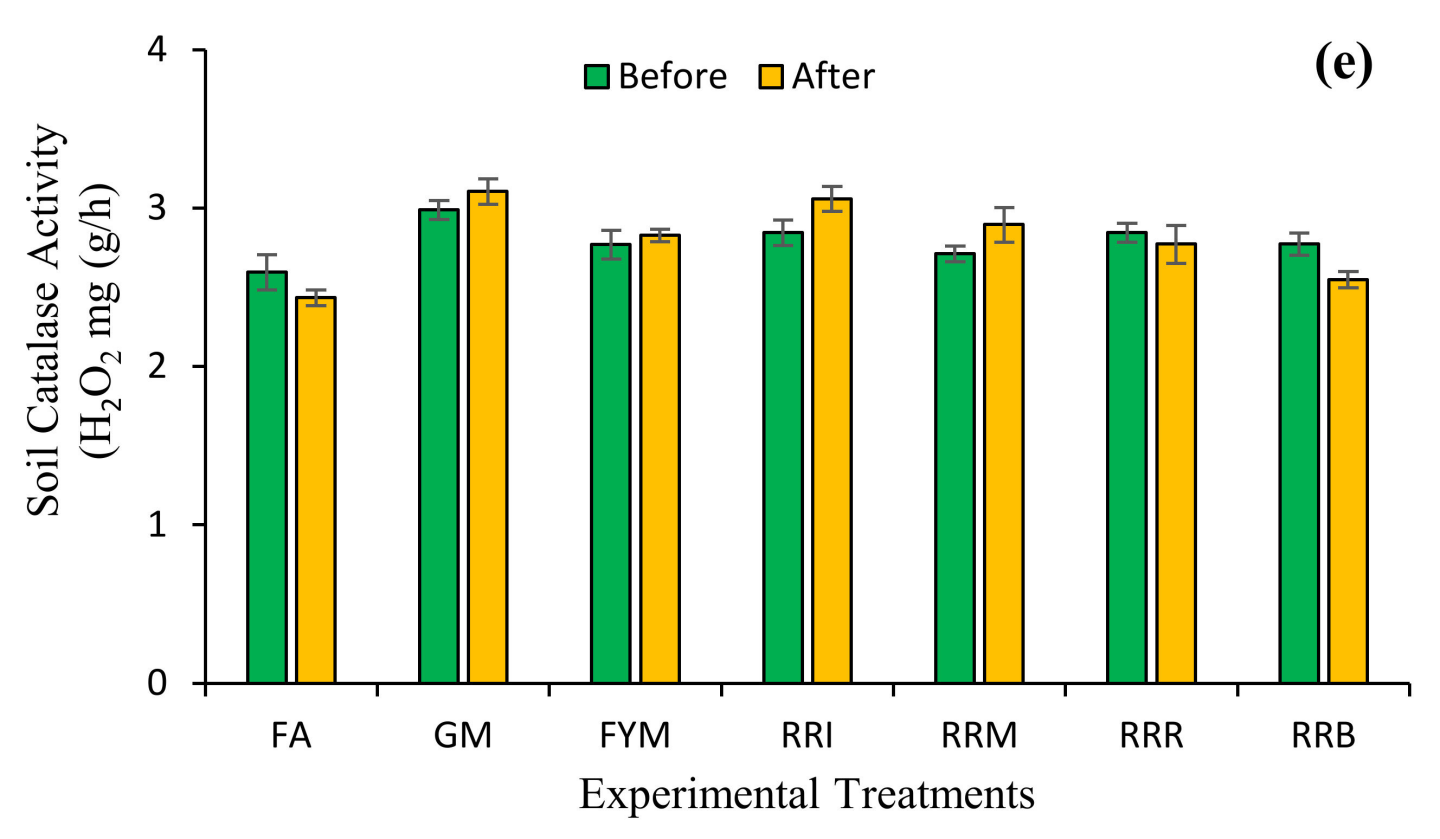
Effect of diverse farming approaches on the enzymatic attributes [soil alkaline phosphatase (**a**), soil dehydrogenase activity (**b**), soil urease activity (**c**), soil invertase activity (**d**), and soil catalase activity (**e**)] of soil samples by fertilizer application (FA); green manuring (GM); farmyard manure application (FYM); rice-residue incorporation (RRI); rice-residue mulching (RRM); rice-residue removing (RRR) and rice-residue burning (RRB). Bar data are means ± standard error (*n* = 3). In each graph, bar data with different letters indicate statistically significant difference at *p*  ≤  0.05.

There were significant differences in soil enzymatic attributes in soil samples among farming approaches and between sampling times (before and after treatments) (Figure 6). Soil alkaline phosphatase activity in rice-residue removing and rice-residue burning treatments were decreased by 2.66 and 4.31%, respectively. However, fertilizer application, green manuring, farmyard manure application, rice-residue incorporation and rice-residue mulching increased soil alkaline phosphatase activity by 0.05, 2.98, 1.51, 5.19 and 3.75%, respectively. The rice-residue removing and rice-residue burning treatments decreased the dehydrogenase activity of soil by 1.84 and 14.51%, respectively. However, fertilizer application, green manuring, farmyard manure application, rice-residue incorporation and rice-residue mulching increased dehydrogenase activity of soil by 1.58, 3.80, 4.00, 13.76 and 3.96%, respectively. Soil urease activity following fertilizer application, rice-residue removing and rice-residue burning treatments were decreased by 4.95, 9.59 and 7.37%, respectively. However, green manuring, farmyard manure application, rice-residue incorporation and rice-residue mulching increased urease activity of soil by 3.38, 5.39, 8.82 and 5.41%, respectively. However, for soil invertase activity, fertilizer application, rice-residue removing and rice-residue burning decreased the invertase activity of soil by 1.08, 1.09 and 8.99%, respectively. Invertase activity of soil following green manuring, farmyard manure application, rice-residue incorporation and rice-residue mulching treatments was increased by 4.44, 6.00, 15.22 and 8.43%, respectively. Soil catalase activity following fertilizer application, rice-residue removing and rice-residue burning treatments was decreased by 6.16, 2.58 and 8.05%, respectively. However, catalase activity of soil following green manuring, farmyard manure application, rice-residue incorporation and rice-residue mulching treatments was increased by 3.90, 2.17, 7.49 and 6.76%, respectively (Table 3).

## 3. Discussion

Due to urbanization and population pressure, the use of bioresources is considered a potential tool for improving soil health indices in agricultural systems in developing countries [28]. Fertile soils support biological productivity, maintain environmental quality, and promote plant and animal health. However, intensive cropping, imbalanced fertilization, poor water management, and frequent ploughing have led to a serious decline in soil fertility, which threatens crop productivity [29].

The findings of the current investigation has revealed that well-decomposed farmyard manures (FYM) contain a score of important primary and secondary micronutrients which play a significant role in improving soil health and crop growth. However, nutritional composition of farmyard manures is highly dependent on different factors including its origin, composting time, temperature of heap, ripening period, addition of microbes and rainfall [30]. Moreover, nutrient contents present in FYM also depend upon the fodder type consumed by dairy animals [31,32], bedding material from crop, crop residues, labour skills and other climatic factors [33].

In the current study, variation in the nutrient concentrations of the sesbania crop might be attributed to different climatic and edaphic conditions [34]. However, some researchers have also reported that variation in the nutrient status of any crop also depends upon the fertilizer plan or the organic and inorganic amendments that were applied to the preceding crop [35,36]. In the current study, sesbania was used as a green manure crop primarily because of its higher nutritional concentration which might be useful for sustainable supply of nutrients to the soil [37]. Similarly, variation in the nutrient concentration of rice cultivars might be due to differences in genetic source, edaphic conditions, cropping system and climatic conditions [38,39,40]. Pedigree selection and genetic variations among cultivars might be the reason for variation in nutrient contents in the rice straw of basmati rice cultivars [28]. Furthermore, the concentration of different biomolecules may vary in different plant organs, especially grains, straw and roots of crop species, tissue type and phenological stage of the crop [41,42].

The results of the current study further revealed that the diverse farming approaches significantly affected the biological and enzymatic attributes of the soil. However, soil physical attributes were not affected by the studied farming approaches. Some studies have reported that residue managements do not have impact on soil physical properties [43,44,45,46]. However, minor changes in the percentage of sand, silt and clay might be due to the source of irrigation water [47,48,49]. Furthermore, the effect of residue management on soil bulk density (BD) was found to be variable, as some researchers reported no effect [50,51], whereas others found lower soil BD in a conservation tillage-residue management system [52], residue incorporation [53], and no tillage surface-residue treatments compared to control [29,54,55]. Moreover, the addition of FYM, green manures and rice straw management had been associated with greater water-holding capacity of soils and crop yields [27,56,57]. Similarly, the application of chemical fertilizers increased the bulk density of soil up to a certain extent [58,59]. However, in some cases, application of NPK fertilizers reduced the bulk density of soil, which might be due to enhanced production of plant biomass, with a consequential upsurge in organic matter content of the soil [59].

The current research further showed that incorporation of organic amendments such as residues, green manuring and FYM increased soil pH to a certain extent. However, EC was decreased, which might be due to decarboxylation of organic anions [29,60,61,62]. Many studies have reported an increase in soil pH irrespective of whether crop residues were burnt, incorporated, or mulched [63]. An increase in pH and EC after burning was generally attributed to ash accretion, which is generally dominated by carbonates of alkali and alkaline earth metals [29,64].

Soil organic carbon (SOC) sequestration significantly contributes to the improvement of soil fertility [65]. However, chemical fertilizers stimulate organic matter decomposition in soil which ultimately results in reduction in total SOC and N in soil, possibly because of higher N uptake by crop plants or leaching [66]. Accumulation of SOM in soil is a reversible process, and the intensive rice–wheat cropping system is responsible for its reduction in agroecosystems [67]. However, this reduction in the concentration of SOM can be compensated for by the incorporation of different crop residues in soil, which help soils to regain their lost fertility [68,69,70,71]. The burning of certain cereals also resulted in higher P and K contents in the soil surface [18,29,54]. The addition of green manure crops and FYM not only improved SOC, nutrient availability and intake of nutrients by crops, but also enhanced the yield of succeeding crops [72,73]. Moreover, green manure crops and FYM limit nutrient leaching, and also mitigate the harmful effect of agrochemicals and soil-borne phytopathogens in the soil [74,75,76].

It was also observed in the current study, that addition of rice residues (incorporation and mulching), green manuring and FYM increased the soil’s bacterial and fungal populations when compared to chemical fertilizer application, rice-residue removal and rice-residue burning treatments. The decomposition of green manures and FYM favours microflora by providing both C and energy for growth and formation of new cell material, which further multiplies the soil microbial community in the decomposing OM, resulting in maximum amounts of microfauna in the soil ecosystem [77]. However, the application of chemical fertilizers affects microbial diversity in a number of ways [78]. All NPK fertilizers hinder the growth of mycorrhizal fungi, but the extent of such hindrance is dependent on the fungal species and the soil available P level [79,80]. Crop residues provide substrate and C and N for growth and activities of soil microorganisms [70], however, these organisms also compete with plants for available nutrients, including those released from residues by decomposition [81,82,83]. Soil microbial biomass (SMB) is affected by the residue management practices and a significant decline in microbial biomass was observed when residues are burnt [29,54,84,85,86].

**Figure 7:**
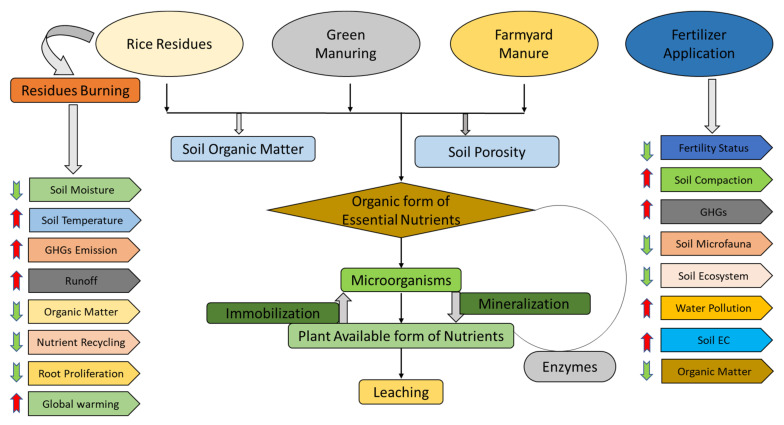
Rice-residue management interventions along with organic manures such as farmyard manure and green manure improves soil physicochemical, biological and enzymatic conditions. More microbial activity was noticed with residue incorporation than with residue removal or burning. Furthermore, sole fertilizer application not only disturbs the soil physicochemical, biological and enzymatic attributes but also causes GHG emission, resulting in global warming.

Findings of the current investigation showed that diverse farming approaches significantly influenced the soil enzymatic attributes. Fertilizer application, rice-residue removal and rice-residue burning decreased the enzymatic attributes. However, rice residue incorporation, FYM and green manuring practices improved soil enzymatic attributes. The observed increase in enzymatic activities due to green manure and FYM amendments is in accordance with previous studies [87,88,89,90,91]. A possible mechanism by which crop-residue incorporation affects soil enzymatic activities is by changing the physicochemical characteristics of soil to influence soil enzyme activity [92,93] (Figure 6). Similarly, Kotroczó et al. [94] observed that soil enzyme activity is stimulated by root reactions. The surface of fresh residues can provide substrates for enzymatic reactions and stimulate plant roots to exude enzymes into the soil. This is also possibly related to the soil temperature; however, further verification of this relationship is required [95]. In this study, the treatments with rice residues increased soil invertase activity more than the fertilizer-alone treatments, which is consistent with changes in SOC. Therefore, we hypothesize that soil enzymatic activities increase with the higher SOC caused by residue incorporation (Figure 7).

Foster et al. [96] reported similar findings for C-cyclase enzymes, which are also related to the colocalization and stability of C substances and enzymes on the surface of rice residues. Since the application of crop residues accelerates N mineralization, an increase in N-cycling enzyme activity occurs [97]. The highest activity of phosphatase among the three rice-straw modifications (incorporation, removal and mulching) varied with the rice growing season and was different from patterns of invertase and urease activity. Similarly, alkaline phosphatase activity may be affected by many factors, such as soil pH and soil moisture [98]. Criquet et al. [99] confirmed that phosphatases may be generated by bacteria or other microorganisms. However, due to the interference of high levels of phosphatase activity originating from roots, it is difficult to study the kinetics of microbial phosphatase production in soil.

## 4. Materials and Methods

### 4.1. Experimental Site

**Figure 8:**
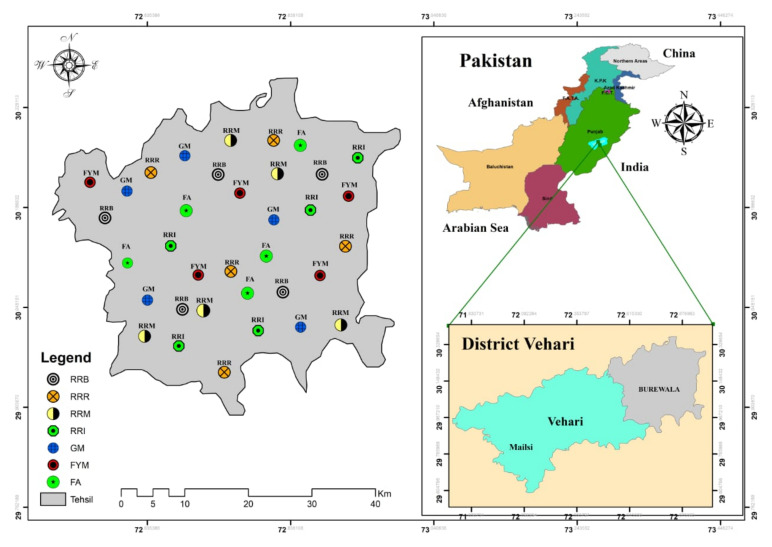
Study area map indicating all the sampling points with diverse farming approaches in the experimental site.

The experimental sites located in Tehsil Burewala, South Punjab Pakistan, lie between 30°10′ and 31°22′ north latitude and 72°39′ and 73°55′ east longitude, with an elevation of 148.4 m above the sea level. The climate condition of the region is arid, with a maximum average temperature of 22.7 °C and minimum average temperature of 5 °C. Average rainfall is 255 mm (Pakistan Meteorological Department, PMD). The main crops are wheat, rice and cotton. Additionally, sugarcane, maize and pulses are also grown in minor quantities (Figure 8).

### 4.2. Experimental Design and Treatments

**Figure 9:**
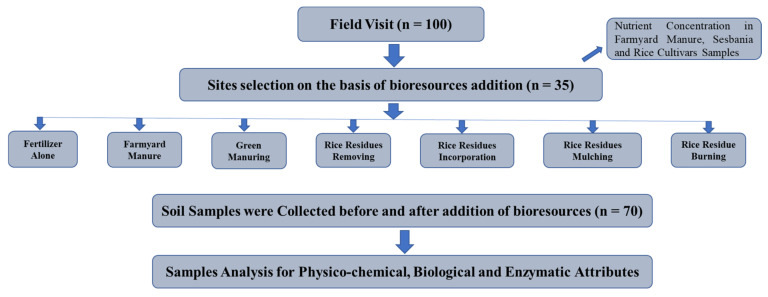
Overview of the experimental design and activities during course of study.

Seven farming approaches: chemical fertilizer alone, green manuring, farmyard manure application, and rice-residue mulching, incorporation, burning, and removing, were selected under observation for determination of soil health attributes. These farming practices were selected following the analysis of a field survey (100 farmers), which observed each farming approach that the residents were practicing on regular basis in their field area for the last three years, on rice–wheat cropping systems. The experiment consisted of the seven farming approaches and five replicates for each treatment, giving a total of 35 sites and was repeated in three continuing years (Figure 9). Soils at 0–20 cm depth were sampled from each site prior- and post- treatment, for analysis of physiochemical and biological properties (such as enzymatic activities) in the soils. Farmyard manure and green manure (Sesbania) (in triplicate) were collected to characterize nutrient concentrations in the straw of five rice cultivars (e.g., Super Basmati, Kissan Basmati, Kianat Basmati, Punjab Basmati and NIAB Basmati).

### 4.3. Elemental Analysis of Rice Residues, Sesbania and Farmyard Manure

Elemental analyses of triplicate samples of rice straw (2–3 g) of potential rice cultivars, green manure crop (Sesbenia) (2–3 g) and farmyard manure (from cattle, composted for 1 year, oven dried) were conducted following the protocols of Norton et al. [100]. Briefly, dried samples of each bioresource were ground and digested using HNO_3_ + HCl (3:1) and H_2_O_2_ on a block digester, with H_2_O_2_ used to enhance the oxidation process. Elemental analysis was performed by inductively coupled plasma mass spectroscopy (Agilent 720 ICP-OES). The internal standard containing 10 μg/L indium was used. The concentration of nitrogen was obtained by analysing the powdered samples using an NCS analyser (NA2500 Elemental Analyzer, UK).

### 4.4. Measurements of Physicochemical and Biological Properties in Soil

Soil samples were randomly collected at depths of 0–20 cm from each site using a soil auger, before and after practicing the specific farming approach. The samples were placed into labelled plastic bags. Collected soil samples were air dried, ground and sieved (2 mm sieve). The samples were divided into two parts for analysis of physicochemical and biological properties and for microbial culturing and enzymatic activities, respectively.

Soil textural class of the collected samples was determined by hydrometer method 101. Dried and sieved soil samples (50 g) were added into a 500 mL beaker and then mixed with of 70 mL of Na_6_[(PO_3_)_6_] 2% solution as a dispersion agent. Before the process of incubation (20 °C for 24 h) to facilitate the process of dispersion, 150 mL of distilled H_2_O was added into the beaker. The suspension was stirred for 15 min on mechanical shaker and then transferred to graduated cylinder. After shaking with metal plunger, an initial reading was observed after 40 s and a final reading was noted after 4 h with a hydrometer [101]. After that, soil textural class was determined using a texture class triangle. Soil suspensions from filtrates were measured for pH and electrical conductivity with a pH meter and an EC electrode, respectively [102].

Soil to water was mixed at a ratio of 1:1 on a mechanical shaker for 20–30 min. The suspension was filtered through 40 μm of filter paper. Separated soil samples were collected to determine soil bulk density (BD) after being dried in an oven at 104 °C. Soil bulk density (BD) was calculated using the following formula: BD (g/cm^3^) = W/V; where W = soil weight (g); V = volume of soil sample (cm^3^).

Kjeldhal’s method was used for analysing total nitrogen in the soil samples, and the vanadomolybdate method for available phosphorus [103], the flame photometer method for extractable potassium [104], and the spectrophotometer method for available Zn, were used, respectively. For soil organic matter contents, the method of Loss of Ignition (LOI) was used [105]. Soil organic matter (SOM) percentage and loss of ignition (LOI) were calculated by using the given equation [106]. LOI% = SOM% = (M_105_ − M_550_/M_550_) × 100, where M_105_ is weight of 10 g of the soil sample after 2 days of air drying and then oven-drying for 24 h at 105 °C to overcome the moisture in the soil, while M_550_ is weight of 1 g soil after taking it into muffle furnace at 550 °C for 5 h in a crucible.

The dehydrogenase activity of soil was determined following the protocols of Min et al. [107]. The alkaline phosphatase activity of soil was calculated using a spectrophotometer as defined in Tabatabai and Bremner, [108]. The soil urease activity (NH_4_-N mg/(g/2h/37), which explains the nitrogen in ammonium form, was analysed for 2 h at room temperature based on the method of Dick, [109], the soil invertase activity (glucose mg/(g/24h/37), which explains the glucose that is produced, was analysed over 24 h at room temperature as described by Frankeberger and Johanson [110], and the soil catalase activity (H_2_O_2_ mg (g/h), which explains the amount of hydrogen peroxide that catalase can decompose, was analysed over 1 h using the method of Johnson and Temple, [111].

Microbial colonies (bacteria and fungi) were assessed using the method of sequential dilutions by spiral plating on agar plates. Half-strength R2A agar plates were used for the estimation of total amount of culturable bacteria [112,113,114], and culturable fungi was assessed by dextrose agar of bengal rose potato [115]. After 48 h of cultivation, clonal population tests were conducted.

### 4.5. Statistical Analysis

Collected data (3 years pooled) were analysed using “Statistix 10” statistical packages. Data regarding analytical analysis of rice residues and green manure crop were analysed using descriptive statistics. All the treatment means were analysed at a significance level of *p* < 0.01 by two-way ANOVA and highest significant difference (HSD) test. Graphical work was performed using Microsoft Office software (Version, 2016).

## 5. Conclusions

Inclusion of rice residues and green manure crops, such as sesbania and farmyard manure, improved soil health, which is critical to the persistence and sustainability of agricultural systems. The diverse farming approaches used in this study significantly enhanced the physicochemical, biological and enzymatic attributes of soil. Organic fertilizers (FYM) and green manure (GM) improved more indicators related to soil health than chemical fertilizer input alone. Rice crop residues are highly siliceous, and have the potential of transforming electrochemical properties of acidic soils that reduce P fixation; thus improving base retention and increasing the soil pH. In this study, rice-residue management interventions and all other diverse farming approaches improved soil physicochemical, biological and enzymatic conditions. Residue incorporation resulted in more microbial and enzymatic activity than fertilizer application alone, residue removal or burning approaches. Using diverse farming approaches will help improve the physical, chemical, and biological indicators of soil health and the consequent provisioning of agroecosystem services. By adopting these strategies, we can achieve the sustainability goals for climate change mitigation, adaptation, impact reduction and early warning.

## Figures and Tables

**Table 1:** Nutrient Concentration in Farmyard Manure Samples and Sesbania Samples (Means ± SE).

Nutrient	Unit	Farmyard Manure	Sesbania
Nitrogen	%	0.91 ± 0.04	3.32 ± 1.04
Phosphorous	%	0.36 ± 0.02	0.73 ± 0.09
Potassium	%	0.89 ± 0.05	1.32 ± 0.15
Calcium	%	0.90 ± 0.03	1.34 ± 0.13
Magnesium	%	0.20 ± 0.04	208.00 ± 3.74
Sulphur	%	0.02 ± 0.03	0.20 ± 0.12
Sodium	%	0.09 ± 0.03	-
Zinc	mg g^−1^	56 ± 1.23	-
Iron	mg g^−1^	140.30 ± 3.42	-
Boron	mg g^−1^	2.30 ± 0.97	-
Copper	mg g^−1^	2.80 ± 1.11	-
Manganese	mg g^−1^	69.00 ± 1.32	-

**Table 2:** Percentage change in the physicochemical attributes and nutrient concentrations in the soil samples with diverse farming approaches.

Treatments	pH	EC	N	P	K	OM	Zn	Bacteria	Fungi
FA	1.85	11.42	21.43	−0.47	0.25	−5.88	0.60	1.43	0.00
GM	1.78	−6.91	35.00	7.59	3.18	7.65	10.34	3.90	0.00
FYM	1.40	−5.28	46.67	5.43	3.13	6.84	12.57	9.21	9.09
RRI	−5.61	−8.33	36.36	12.34	2.79	17.11	24.46	11.84	9.09
RRM	−2.40	−2.17	8.33	3.39	2.42	6.93	4.55	6.67	0.00
RRR	−1.74	−3.11	−8.00	−0.38	−2.24	−3.93	−1.15	−2.67	0.00
RRB	−2.50	11.05	−22.22	−6.79	4.44	−17.42	−7.91	−9.59	−20.00

EC = Electrical conductivity; N = Total Nitrogen Contents; P = Available Phosphorous Contents; K = Extractable Potassium Contents; OM = Organic Matter; Zn = Available Zinc Contents.

**Table 3:** Percentage change in the enzymatic attributes of soil samples following diverse farming approaches.

Treatments	AP	DH	Urease	Invertase	Catalase
FA	0.05	1.58	−4.95	−1.09	−6.16
GM	2.98	3.80	3.38	4.44	3.90
FYM	1.51	4.00	5.39	6.00	2.17
RRI	5.19	13.76	8.82	15.22	7.49
RRM	3.75	3.96	5.41	8.44	6.76
RRR	−2.66	−1.84	−9.59	−1.09	−2.58
RRB	−4.31	−14.51	−7.37	−9.00	−8.05

AP = Alkaline Phosphatase Activity; DH = Dehydrogenase Activity.

## Data Availability

Data is contained within the article.
